# Exploration and practice of data-driven high-quality development in smart hospitals

**DOI:** 10.3934/publichealth.2026015

**Published:** 2026-03-02

**Authors:** Lijuan Li, Chuanzi Yang, Zhiyan Zhang, Qiang Wu, Xiaojun Cao

**Affiliations:** Guangzhou Women and Children's Medical Center, Guangzhou Medical University, Guangzhou, Guangdong, 510623, China

**Keywords:** data-driven, high-quality development, smart hospitals, clinical decision support system, intelligent management

## Abstract

**Background:**

In this study, we explored a data-driven approach to smart hospital construction, addressing challenges such as data silos, fragmented processes, and delayed decision-making within healthcare systems. The proposed framework supports the high-quality development of public hospitals in China and facilitates intelligent integration among healthcare delivery, service provision, and management functions, achieving synergistic advancement of the “trinity” of hospital operations.

**Method:**

A phased action research method was employed, comprising three stages: (1) Infrastructure construction period (2016–2018): Achieving system interconnection and data sharing based on enterprise service bus, standardized interface transformation, and the establishment of an information security system. (2) Intelligent empowerment period (2019–2021): Implementing intelligent healthcare processes, patient services, hospital management, and closed-loop management. (3) Ecological expansion period (2022–2024): Constructing a regional pediatric alliance data collaboration network and enabling internet hospital service continuity. The core pathways include global data integration, data governance standardization, closed-loop management enhancement, intelligent decision support development, and internet hospital integration.

**Results:**

The study demonstrated four outcomes: (1) Improved data quality: Standardization, completeness, and consistency were significantly improved (*p* < 0.001). (2) Enhanced medical quality: The accuracy rate of AI-assisted diagnosis exceeded 90%, the completion rate of clinical pathways reached 96.03%, and the intensity of antibiotic use (defined daily doses) decreased from 28.27 to 17.14. (3) Optimized service efficiency: The appointment completion rate was 98.15%, the average length of stay in a hospital decreased by 42.06% (interrupted time series analysis showed that the post-intervention level was significantly reduced, *β*_2_ = −0.701, *p* = 0.002), and the number of bed turnover increased by 128.8% (*β*_2_ = 14.558, *p* < 0.001). (4) Significant regional collaboration: Established collaborations with 134 hospitals, with more than 2629 cases of remote consultation and transfer in the year 2024 and 88,900 times of mutual recognition of test results.

**Conclusion:**

The construction of a data-driven smart hospital, through systematic integration and intelligent empowerment, demonstrated positive outcomes in improving data quality, clinical performance, operational efficiency, and regional collaboration. This provides a replicable implementation framework for the high-quality development of public hospitals in China. Since this was a single-center exploratory study, subsequent multi-center validation is necessary. Continuous improvement of data governance and comprehensive regulatory systems is required to further advance medical services toward enhanced precision, collaboration, and intelligence.

## 1. Introduction

Guided by the “Healthy China 2030” strategy [1], the “Opinions on high-quality development of public hospitals” explicitly proposed “strengthening the supporting role of informatization and promoting the upgrade of smart hospital construction” [2]. This requirement has deepened into a top-level framework for the synergistic development of the “trinity” of smart healthcare, smart service, and smart management. The National Health Commission's 2025 “Medical Health Big Data Application Action Plan” further requires “building a data-driven hospital intelligent decision-making system” [3], signaling a new stage in medical digital transformation: A transition from system integration to the release of data value. International trends indicate that the healthcare sector is undergoing a paradigm shift fueled by the convergence of big data, Artificial Intelligence (AI), and the Internet of Things (IoT), with researchers aiming to build more efficient, personalized, and accessible smart healthcare systems [4]–[8].

Domestic and international research predominantly focuses on technologies (e.g., machine learning and IoT), single domains (e.g., AI-based imaging diagnosis and disease prediction), or theories (e.g., data privacy protection) [4],[9]. However, there is a lack of detailed case studies and process evaluations on how large medical institutions can systematically, and in phases, implement end-to-end, data-driven transformation encompassing data integration, governance, application, and ecological expansion [5]. Furthermore, during smart hospital construction, data silos, process gaps, and decision-making delays in hospital management applications pose key structural challenges.

Therefore, we conducted, from year 2016 to 2024 at the Guangzhou Women and Children's Medical Center (four campuses), a longitudinal action research project. We explored and evaluated the implementation of a multi-stage, data-driven framework for large-scale smart hospital construction and assessed intermediate outcomes. We aimed to provide practical experiences and insights to support the high-quality development of public hospitals in China.

## 2. Materials and methods

### 2.1. Research design and context

We employed a phased action research method [10]. Each stage followed an iterative “plan–action–observe–reflect” cycle, conducting research while promoting change, constituting a single-center, phased, and before-and-after intervention comparative quality improvement study. The research period spanned 2016 to 2024, divided into three stages: A foundational construction period (2016–2018), intelligent empowerment period (2019–2021), and ecological expansion period (2022–2024).

### 2.2. Research implementation phase

**Figure 1.**
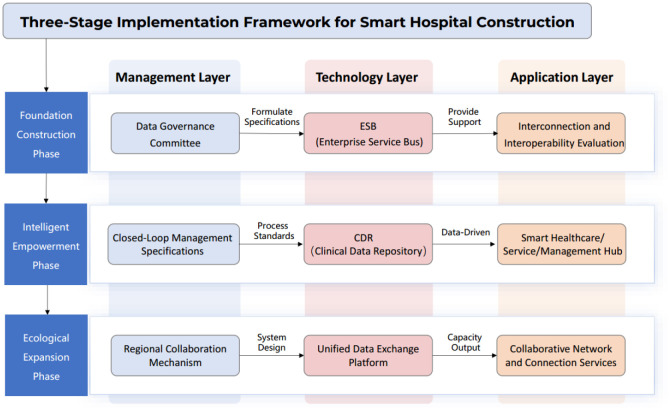
Comprehensive structure diagram of the research framework.

This study was conducted at Guangzhou Women and Children's Medical Center, which handles over 5 million outpatient and emergency visits annually and over 160,000 hospitalizations, possessing a relatively solid informatization foundation. We comprehensively covered the three implementation stages of data-driven smart hospital construction. Each stage was structured according to the core logic of three levels: Management, technology, and application (Figure 1). Specific intervention measures were as follows:

#### 2.2.1. Phase 1: Infrastructure construction period (2016–2018)

In this stage, we focused on platform-based and standardized construction, completing information system integration and security framework establishment.

(1) Shared document standardization: To facilitate cross-system data sharing, we built a Master Patient Index (MPI) system and a shared document repository. Using an Enterprise Service Bus (ESB), the processes of generating and accessing 53 types of shared documents were standardized, enabling seamless data exchange across systems [11].

(2) Service interface transformation and system integration: Based on the “hospital information interconnection standardization maturity assessment scheme (2020 Edition)”, interface specifications were organized. Phased integration of non-integrated and standardized transformation of integrated systems were completed [12].

(3) Information security system establishment: Adhering to national interoperability and information security standards, the organization formulated information security policies and management objectives. We constructed a unified platform for information security management. This platform established a security management system encompassing guiding documents, procedural manuals, and record-keeping materials [13],[14].

#### 2.2.2. Phase 2: Intelligent empowerment period (2019–2021)

In this stage, we focused on addressing business process intelligence and data empowerment.

(1) Intelligent healthcare processes: A closed-loop management system was developed based on the clinical data repository to cover the process of outpatient care, emergency care, hospitalization, and patient transfers between departments. This system integrated critical value alerts, clinical decision support, and clinical pathway management.

(2) Intelligent patient services: Based on the hospital integration platform and mobile applications, a comprehensive intelligent service system covering pre-consultation, consultation, and post-consultation phases was implemented. This system included smart appointment scheduling, online payment, proactive follow-up, and electronic health record management.

(3) Intelligent hospital management: Based on the hospital information platform and IoT system, an integrated “resource–quality–decision” intelligent management hub [15] was constructed, enabling dynamic resource scheduling and data-driven operational decisions.

#### 2.2.3. Phase 3: Ecological expansion period (2022–2024)

In this stage, we aimed to extend service boundaries and build a collaborative healthcare ecosystem.

(1) Constructing a regional pediatric alliance data collaboration network: Utilizing technologies like 5G and the internet, a unified data exchange platform was established. This enabled cross-institution integrated clinical data views, remote consultation collaboration, and convenient referral models between levels of care.

(2) Internet hospital continuity of service: Based on the physical hospital's internet hospital platform, it provided “follow-up–management–rehabilitation” full-cycle cloud-based medical closed-loop services for discharged patients and individuals with chronic conditions.

### 2.3. Core implementation pathways

The smart hospital construction process was essentially a transformative process in which the value of data was continuously discovered, governed, and applied. Data, as the key production factor, flowed along the primary process of domain-wide collection and aggregation, unified governance and standardization, intelligent enablement and application, and ecological collaboration and extension. This process ran through the three stages of foundational construction, intelligent enablement, and ecological expansion. The core implementation pathways of this study revolved around this primary process, manifested in the following five interrelated and progressive aspects:

#### 2.3.1. Global data integration

With the primary goal of breaking down “data silos” and achieving interoperability, a hospital information integration platform was built relying on an ESB [16]. This platform, serving as the “central nervous system”, a metaphor highlighting its role in coordinating and controlling various systems, connected 68 core business systems, such as Electronic Medical Record (EMR), Laboratory Information System (LIS), Picture Archiving and Communication System (PACS), and Hospital Information System (HIS), covering clinical, management, and service domains through standardized adapters. This setup enabled real-time collection and synchronization of patient data across all business nodes, spanning admission through to discharge. Building on this foundation, unifying data models and standards was essential: Establishing a hospital-wide Enterprise Master Patient Index (EMPI) ensured unique patient identity, and strict adherence to the Health Level Seven (HL7) Fast Healthcare Interoperability Resources (FHIR) international standard [17] defined 53 types of standardized shared documents. This provided structurally unified and semantically consistent raw data for cross-system data exchange and subsequent deep utilization.

#### 2.3.2. Data governance standardization

High-quality data is the foundation for credible analysis. Drawing on the international Data Management Association (DAMA) data governance framework, we constructed a three-tier governance system comprising management, technology, and application levels [18]. The management tier established a data governance committee, formulated unified standards, and clarified data ownership and responsibilities. The technology tier leveraged the data platform and utilized standards like FHIR to clean, transform, and standardize coding; for example, unifying different versions of International Statistical Classification of Diseases (ICD), on multi-source heterogeneous data. This process systematically improved data standardization, completeness, and consistency. The application tier integrated governance rules into business processes, incorporated indicator systems to monitor performance, and implemented routine data quality control through dynamic monitoring and alerts. Together, these tiers ensured that data captured from the source was trustworthy and usable.

#### 2.3.3. Closed-loop management enhancement

Well-governed, high-quality data was applied to core medical processes to enhance quality and safety. First, a systematic implementation of end-to-end electronic closed-loop systems covering 20 key processes such as blood transfusion, medication, and critical laboratory values was achieved. Through system-enforced rules and barcode scanning via mobile devices, traceability of processes and prevention of errors at various stages of the process were ensured. Second, the digitalization of clinical pathways was advanced. A total of 121 categories were incorporated into standardized pathway management. Utilizing a rules-based quality control system, real-time monitoring and feedback on deviations from the pathway were provided. This drove the transition of clinical practices from experience-driven to data-driven standard approaches.

#### 2.3.4. Intelligent decision development

In the hospital management domain, data value lies in real-time perception and intelligent decision-making. By integrating resource status data, including equipment, supplies, and beds, through IoT technology, we constructed an integrated operational decision-making dashboard that enables real-time dynamic monitoring of core indicators such as efficiency and cost. Building on this integrated dashboard, we introduced machine learning algorithms for big data analysis, which enable prediction and flexible scheduling of resource demands, including beds and equipment. This advancement promoted an upgrade in the management model from post-hoc statistical analysis to a closed-loop intelligent management cycle: “Monitoring–alerting–intervention–optimization”.

#### 2.3.5. Internet hospital integration

The value of data is amplified in service collaboration. Internally, the internet hospital platform bridged online and offline services, integrating data from appointment, payment, medication delivery, etc., to build a patient-centered seamless service closed-loop, improving accessibility and experience [19]. Externally, utilizing technologies like 5G, a regional collaboration network was constructed. Under the premise of security and compliance, cross-institution data flow and business collaboration for results mutual recognition and remote consultations were achieved, promoting the evolution of healthcare services from isolated intra-hospital scenarios to an integrated regional ecosystem.

### 2.4. Evaluation method

The smart hospital construction is a long-term systematic endeavor. It has delayed and cumulative effect manifestations, making it difficult to fully measure with static indicators at a single point in time. Therefore, we adopted a mixed-method evaluation strategy, combining process evaluation and outcome evaluation. Focusing on the core data-driven approach, we constructed a comprehensive evaluation system covering four dimensions: Data foundation, medical quality, service efficiency, and regional collaboration. All quantitative data used for evaluation were anonymized and extracted from core business systems such as the hospital data integration platform to ensure authenticity and traceability. Data sources, variable definitions, and evaluation indicators are described below.

#### 2.4.1. Evaluation indicators and definitions

Evaluation indicators closely align with the data value chain of smart hospital construction. Indicator definitions are as follows:

*Data quality:* Data quality, a key intermediate outcome reflecting data governance effectiveness, is assessed monthly via stratified random sampling and includes three core indicators [19].

(1) Standardization: The proportion of data elements conforming to established data standards (e.g., diagnosis codes conforming to the ICD-10 standard, data format standardization, unification of standardized terminology dictionaries, and complete standardized mapping).

(2) Completeness: The proportion of non-missing fields among mandatory data fields.

(3) Consistency: The proportion of records satisfying cross-field or cross-system logical consistency rules (e.g., compliance with cross-field logic validation and consistency in matching same-name fields across systems).

The calculation formula for each indicator is: Indicator value (%) = number passing inspection / total inspected × 100%.

*Clinical quality:* Clinical quality reflects the effect of data empowerment on clinical decision-making and process standardization. Core clinical quality evaluation indicators include: (a) AI-assisted diagnostic accuracy: Concordance rate between AI diagnostic suggestions and confirmed diagnoses made by senior physicians. (b) Clinical pathway management rate: Proportion of discharged patients meeting clinical pathway admission criteria who were managed under clinical pathways during the specified year. (c) Clinical pathway completion rate: Proportion of patients managed under clinical pathways during the specified year who successfully completed the full clinical pathway process. (d) Antimicrobial consumption intensity (Defined Daily Doses, DDDs): Total consumption of Antimicrobial drugs per 100 patient-days during the specified year.

*Service efficiency:* Service efficiency reflects the outcome of data-driven operational management optimization. Service efficiency indicators are calculated annually: (a) Average length of hospital stay = total hospital bed-days / number of discharged patients during the same period. (b) Bed turnover rate = number of discharged patients during the same period / average number of open beds.

*Regional collaboration:* Regional collaboration evaluates the effectiveness of data circulation and application in a cross-institution ecosystem. Regional collaboration evaluation focuses on quantitative indicators, mostly including: (a) Annual remote consultation volume (unit: case-times) and (b) annual volume of mutual recognition of laboratory and imaging results (unit: item-times). Relevant data are all sourced from the regional medical collaboration platform.

#### 2.4.2 Statistical analysis methods

*Data quality comparison:* Data from 12 months before and after the intelligent empowerment stage (the core intervention stage) were paired and compared. Based on the data distribution, the paired t-test or Wilcoxon paired signed-rank test was used, with the Holm method applied to adjust for comparisons. Descriptive statistics were presented as mean ± standard deviation (M ± SD). The distribution of paired differences was evaluated using Q-Q plots and histograms. All analyses were conducted in language R. A two-sided *p*-value of less than 0.05 was considered statistically significant.

*Service efficiency trend analysis:* Interrupted Time Series (ITS) analysis [20] was employed. This was a quasi-experimental design used to evaluate the temporal trend impact of interventions when random control groups cannot be established, suitable for the long-term observational data of this study.

Annual data for average length of stay and bed turnover rate were systematically collected for 12 years before and after the intervention point. The intervention point was defined as year 2017, with the pre-intervention period defined as year 2013–2016, and the post-intervention period as year 2017–2024. ITS models (Y = *β*_0_ + *β*_1_X_1_ + *β*_2_X_2_ + *β*_3_X_3_ + *ε*) were constructed separately for the two evaluation indicators. This method was suitable for long-term observational data where random control groups could not be established, effectively assessing changes in temporal trends before and after intervention implementation. In the model: Y was the dependent variable (i.e., the evaluation indicator); *β*_0_ was the constant term (the level of the evaluation indicator at the study start); *β*_1_ was the pre-intervention slope (pre-intervention trend); X_1_ was the time sequence (coded sequentially from 1 to 12 by year); *β*_2_ was the level change caused by the intervention; X_2_ was the intervention status (coded “0” for pre-intervention, “1” for post-intervention); *β*_3_ was the change in slope before and after the intervention, where *β*_1_ + *β*_3_ represented the post-intervention slope (post-intervention trend); X_3_ was the post-intervention time sequence (coded “0” for pre-intervention, and sequentially 1 to 8 for post-intervention); and *ε* was the random error term. The Durbin-Watson statistic was used to test for autocorrelation. If autocorrelation was present, generalized least squares with a first-order autoregressive [AR(1)] covariance structure was used for correction. Mainly reported was the level change (*β*_2_) along with its 95% confidence interval and *p*-value.

*Descriptive statistical analysis:* Other metrics were described using frequency, percentage, mean, and other appropriate measures.

## 3. Results

### 3.1. Improved data quality

**Figure 2.**
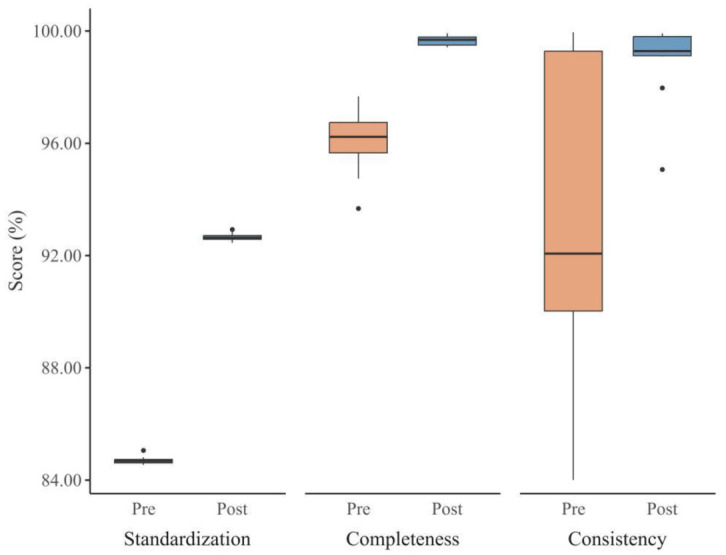
Monthly data quality indicators pre- and post-intervention.

Scores for standardization, completeness, and consistency were higher in the post-intervention period than in the pre-intervention period. Standardization was 84.70% ± 0.14% pre-intervention and 92.66% ± 0.15% post-intervention (95% CI 7.86–8.07; t(11) = 171.85, *p* = 3.25 × 10^−20^). Completeness was 96.11% ± 1.14% pre-intervention and 99.65% ± 0.18% post-intervention (95% CI 2.79–4.30; t(11) = 10.33, *p* = 5.31 × 10^−7^). Consistency was also higher in the post-intervention period (95% CI 2.23–8.55; V = 72, *p* = 1.08 × 10^−2^). Holm-adjusted sensitivity checks for multiplicity across the three indicators did not change these conclusions. Details are shown in Table 1 and Figure 2.

**Table 1.** Comparisons of data quality pre- and post-intervention.

Indicator	Pre (n = 12) M ± SD	Post (n = 12) M ± SD	Paired difference (95% CI)	Test Statistic	*p*-value
Standardization	84.70 ± 0.14	92.66 ± 0.15	7.97 (7.86–8.07)	171.85	3.25×10^−20^
Completeness	96.11 ± 1.14	99.65 ± 0.18	3.54 (2.79–4.30)	10.33	5.31×10^−7^
Consistency	93.41 ± 5.23	98.99 ± 1.34	4.95 (2.23–8.55)	72.00	1.08×10^−2^

Note: Paired differences are reported as mean (paired t-test) or Hodges–Lehmann estimate (Wilcoxon signed-rank test), with 95% CIs. Test statistics are t (paired t-test) and V (Wilcoxon signed-rank test).

### 3.2. Enhanced healthcare quality

#### 3.2.1. AI-assisted decision support

In terms of clinical decision support, we utilized de-identified historical imaging data from the hospital, strictly adhering to privacy protection and ethical review requirements. We employed a deep convolutional neural network model based on transfer learning to assist in diagnosing eye diseases and pediatric pneumonia, aiming to provide reference suggestions for physicians [21]. A balanced random forest algorithm was used to construct a prediction model for the in-hospital mortality risk of sepsis patients, significantly improving the prediction sensitivity for death cases. Among them, the optimized balanced random forest model could help ICU clinicians make predictions, thereby avoiding delayed treatment for sepsis patients who are at risk of death [22].

#### 3.2.2. Clinical pathway implementation

Focusing on constructing a full-process information closed loop, the closed-loop management covering 20 key medical links was completed. A total of 98 disease types were included in clinical pathway management, with 121 clinical pathways established, including 28 secondary pathways formulated for complex or variant situations. The pathways covered 95% of clinical departments in the hospital. As of year 2024, the clinical pathway management rate of discharged patients was 56.46% (95, 958/169, 964), and the pathway completion rate reached 96.03% (92, 144/95, 958).

#### 3.2.3. Standardized medical behavior

**Figure 3.**
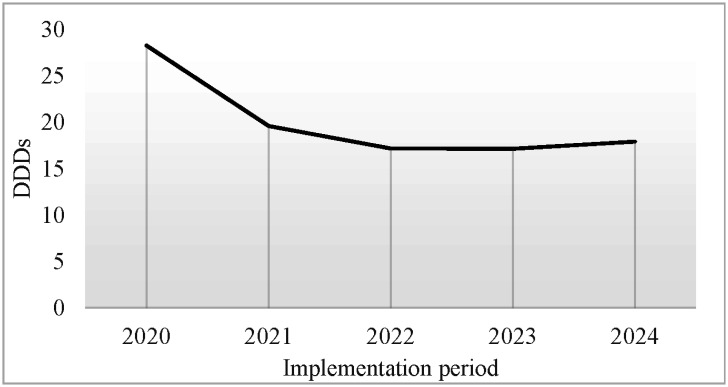
Trend of antibiotic use intensity changes.

Through indicator data monitoring, antibiotic use patterns and issues were analyzed. Standards for rational antibiotic use were formulated, a multidisciplinary review team was established, and scientific management was achieved. The rate of pathogen submission for testing before antibiotic use increased from 51% to 80%, the rate for restricted antibiotics increased to 88%, and the rate for special-grade antibiotics increased to 89.5%. Antibiotic use intensity steadily decreased. DDDs decreased continuously from 28.27 to 17.14. The trend of antibiotic use intensity is shown in Figure 3.

### 3.3. Optimized service efficiency

#### 3.3.1. Optimized appointment rate

Real-name appointment registration was conducted through various channels, including Alipay, WeChat, App, self-service machines, phone, and website. A unified appointment management monitoring and blacklist system was established, enabling orderly time-slot appointment registration and visits. An appointment waiting list mechanism was added. The system monitored appointment slot changes in real-time, reducing repeated refreshing or waiting time. The appointment completion rate increased from 81.14% to 98.15%.

#### 3.3.2. Increased online settlement rate

Medical insurance electronic certificates, aggregated payments options (WeChat/Alipay/ UnionPay), and the hospital information management system were integrated. Payment methods such as mobile payment and self-service machine payment were added, covering over 90% of payment scenarios. Seamless payment pop-ups were embedded at key nodes like registration, prescription generation, examination appointment, and discharge settlement, shortening the settlement path and reducing patient waiting time. In September 2020, online payment for maternity insurance patients was achieved, making the hospital the first in Guangzhou to realize online maternity insurance settlement.

#### 3.3.3. Improved continuity of service efficiency

**Figure 4.**
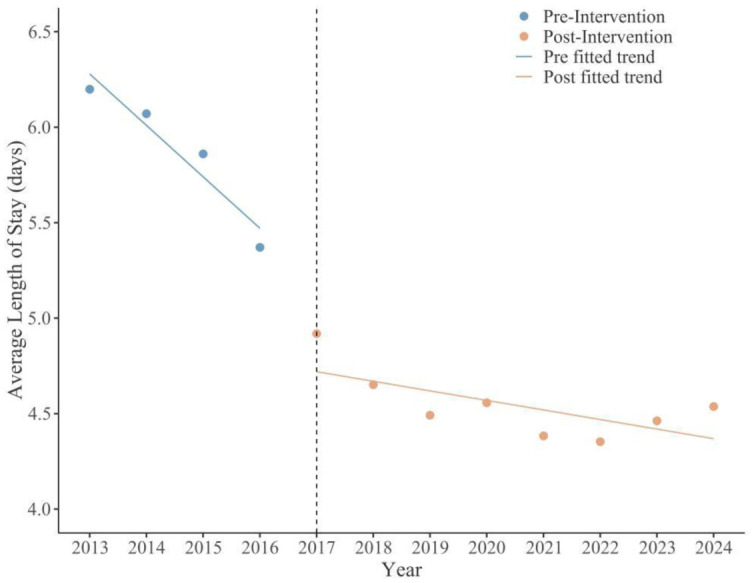
Changes in average length of stay pre- and post-intervention.

**Figure 5.**
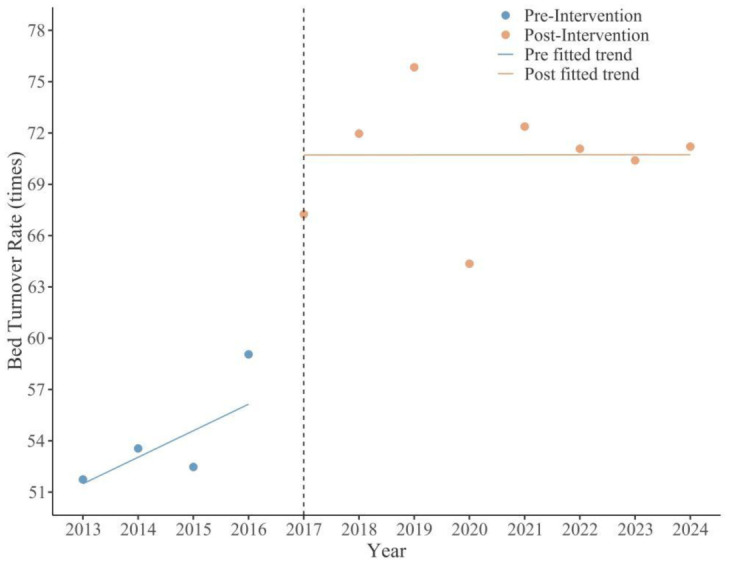
Changes in bed turnover rate pre- and post-intervention.

The results indicated a significant immediate reduction in the average length of stay following the intervention (*β*_2_ = –0.70, *p* = 0.002). The average length of stay decreased from 7.18 days in the pre-intervention period to 4.16 days in the post-intervention period, representing a 42.06% reduction. Concurrently, a significant immediate increase was observed in the bed turnover rate (*β*_2_ = 14.56, *p* < 0.001), rising from 34.65 times to 79.28 times, an increase of 128.8%, enabling limited medical resources to serve more patients and alleviating the “difficulty in hospitalization” problem. Moreover, with the help of the information platform and mobile applications, the discharge follow-up rate significantly increased, achieving timely tracking and intervention of patient recovery status and effectively reducing the unplanned readmission rate. The synergistic optimization of these three key indicators jointly built an efficient continuity of service system covering the cycle of patient “admission–treatment–discharge–rehabilitation”, enhancing the overall efficiency of medical services and patient experience (Figures 4 and 5).

**Table 2.** Regression results from ITS analysis of average length of stay and bed turnover rate.

Metric	Coefficient(*β*)	*p*-value	95% CI	*R^2^*
Average Length of Stay				0.97
*β*_0_	6.55	<0.001	(6.16, 6.94)	
*β*_1_	−0.27	0.002	(−0.41, −0.13)	
*β*_2_	−0.70	0.002	(−1.07, −0.34)	
*β*_3_	0.22	0.010	(0.07, 0.37)	
Bed Turnover Rate				0.88
*β*_0_	49.94	<0.001	(43.4, 56.49)	
*β*_1_	1.55	0.177	(−0.87, 3.96)	
*β*_2_	14.56	<0.001	(8.65, 20.46)	
*β*_3_	−1.54	0.182	(−3.98, 0.89)	

### 3.4. Regional collaboration results

As 2024, the collaboration network encompassed 134 hospitals. The annual number of remote consultations/patient transfers reached 2629 cases, including cross-border transfers to Hong Kong and Macau. Moreover, 5G remote robotic surgery was achieved, contributing to the success rate of pediatric critical care. The number of mutual recognition instances of laboratory and imaging results was 88,900, reflecting the outcomes of the ecological expansion period.

## 4. Analysis and discussion

### 4.1. Major research findings

In this study, through a nine-year longitudinal action research project, we systematically constructed and validated a phased, data-driven smart hospital construction framework. The results showed that the progressive data value chain of “holistic integration—governance standardization—intelligent empowerment—ecological collaboration” is associated with improvements in the healthcare system's data quality, clinical quality, operational efficiency, and regional collaboration capabilities. The significant improvement in data quality; standardization, completeness, and consistency, resulted directly from the combined effects of system integration and standardized governance (e.g., adopting FHIR standards) in the first stage, followed by closed-loop management in the second stage. This provided a solid foundation for subsequent AI-assisted decision-making and precise management.

Indicators such as average length of stay and bed turnover rate hold significant practical importance for smart hospital management. Their improvement likely stems from the following mechanisms working together to enhance overall operational efficiency: (a) Process coordination and optimization: Electronic closed-loop management systems reduce waiting times and errors in diagnosis and treatment processes, thereby accelerating workflows. (b) Resource visualization and intelligent scheduling: The integrated management decision support system makes resource status (beds, equipment, etc.) transparent, facilitating dynamic allocation. (c) Integration of online and offline patient services: Online processes compress non-medical waiting times. These mechanisms work together to enhance overall operational efficiency.

### 4.2. Comparison with the international smart hospital development pathway

Internationally, smart hospital construction often emphasizes technical standardization (e.g., HL7 FHIR) and clinical decision support systems, focusing on data integration through interoperability and leveraging AI to enhance diagnostic and treatment precision [23],[24]. Common implementation pathways are guided by maturity models like healthcare information and management systems society (HIMSS) analytics electronic medical record adoption model (EMRAM) [25], progressively advancing digital closed loops. Our investment in standardization and integration during the foundational construction period, and the deployment of AI-assisted diagnosis during the intelligent empowerment period, align with this global technological paradigm.

Methodologically, international cases often use waterfall models and other project management tools for system construction. In contrast, we employed a phased action research method, treating the construction process as a “management-technical” change process requiring continuous learning and adjustment. Progress is achieved through iterative cycles, enabling dynamic adaptation between the research and practice phases. The phased progression ensures that each construction cycle can be continuously optimized based on feedback, enhancing the adaptability and controllability of the implementation pathway. Regarding expansion logic, the “regional pediatric alliance data collaboration network” constructed in this study, along with the deeply integrated internet hospital services, resonates with international concepts of “value-based healthcare” and “integrated service networks” [26]. However, their advancement momentum is closely coupled with the government-led “hierarchical diagnosis and treatment” system, exhibiting stronger characteristics of administrative coordination and regional homogenized management, differing from the collaboration models in many countries, driven by markets or insurance payers [27].

### 4.3. Generalizability and limitations

In this study, we employed a single-center before-and-after design. Although the observed effects showed a temporal association with the series of interventions, it was difficult to completely rule out potential confounding factors such as concurrent policy changes, industry technological advancements, long-term trends, and regression to the mean. Some analyses had limited sample sizes and lacked parallel controls, which requires further enhancement of the strength of causal inference. However, the construction framework design and the implementation experiences of the digital infrastructure remain valuable for reference. Smaller-scale hospitals or those with weaker digital infrastructure could prioritize focusing on basic data integration and key process closed-loops, advancing gradually. It is important to note that obstetrics/gynecology and children's specialty hospitals, with younger patient demographics and higher digital literacy, more readily accept the online services, mobile applications, and intelligent interactions brought by smart hospital construction. When promoting these services to general hospitals with a higher proportion of elderly patients, emphasis must be placed on bridging the “digital divide”, strengthening aging-friendly adaptations and coordination with offline services, and building an aging-friendly ecosystem of intelligent aids, voice interaction, and remote care services.

## 5. Conclusion

In this study, we systematically describe and preliminarily evaluate, through a 9-year longitudinal case study, the complete process of implementing data-driven smart hospital construction at a national children's regional medical center. The research reveals a digital transformation pathway characterized by three key pillars: Consolidating the data foundation, empowering core processes, and expanding service ecology. This pathway, advanced iteratively in phases, shows a temporal association with positive trends in the hospital's data quality, clinical quality, operational efficiency, and regional collaboration. The implementation logic, process experiences, and intermediate outcomes accumulated in this case not only embody the essence of international standards in technological depth and closed-loop management but also offer concrete reference value for public hospitals in China aiming to promote high-quality development within the framework of the “trinity” smart hospital construction [28].

## Use of AI tools declaration

The authors declare that no artificial intelligence (AI) tools were used in the creation of this article.
